# Use of Pre-Post Treatment Values in Place of Between Group Comparisons Invalidates Meta-Analysis Findings. Commentary: Can Millet Consumption Help Manage Hyperlipidemia and Obesity?: A Systematic Review and Meta-Analysis

**DOI:** 10.3389/fnut.2022.921352

**Published:** 2022-07-22

**Authors:** Marwa Aly, Yasaman Jamshidi-Naeini, Andrew W. Brown

**Affiliations:** ^1^Department of Applied Health Science, Indiana University School of Public Health-Bloomington, Bloomington, IN, United States; ^2^Department of Epidemiology and Biostatistics, Indiana University School of Public Health-Bloomington, Bloomington, IN, United States

**Keywords:** pre-post treatment values, invalidating error, post-publication error correction, meta-analysis, causal inference, between group comparisons

Anitha et al. (1) published a meta-analysis of 19 studies investigating millet consumption on lipid profiles. Upon review of the meta-analysis and its findings, there is an error throughout that invalidates causal inferences in the meta-analysis: the use of pre and post values from only the millet groups in the absence of comparisons between groups. Because of this error, the meta-analysis is unable to answer the question whether millet causally affects lipid profiles with reported findings.

Using pre and post treatment values only measures changes within one group, which will not accurately reflect the causative nature of the intervention because there are many potential variables that have not been controlled. Causal inference in intervention studies is most commonly established by including an appropriate control group. In fact, Anitha et al. (1) included many studies that do have control groups, but they neglected to use control groups in their analyses. Instead, they often extracted baseline values for the millet intervention groups and labeled them as control values in their analysis. For example, we extracted the “control” and “experimental” values from each study as labeled in Figure 5 and compared them to the original articles. In 11 comparisons from nine studies, the “control” values in Figure 5 represent baseline values from the millet group and the “experimental” values represent the follow-up values from the millet group. In one comparison (2), the “control” value is the baseline value of the non-millet group and the “experimental” value is the baseline value of the millet group. Yet, eight of the studies had a comparator group that may have permitted appropriate between-group testing.

Additionally, even if the purpose of the meta-analysis was to investigate within-group changes of lipid profiles after millet consumption, the methods used do not account for within-subject correlations. Pre-post treatment values are not independent of each other. Because pre-post measurements are taken from the same individuals, there is an existing correlation between the values that should be taken into consideration. This correlation can be estimated from published values using appropriate statistical techniques and, when incorporated into the analysis, will affect the overall meta-analysis because the estimated variance and degrees of freedom are affected.

Given the incorrect analytical methods used in this meta-analysis, the evidence presented can neither support nor refute whether millet may benefit the health markers of interest. In situations where there is “clear evidence that the findings are unreliable […] as a result of major error (e.g., miscalculation or experimental error),” the Committee on Publication Ethics suggests editors should consider retracting the publication (3).

## Author Contributions

MA: writing of the first draft. MA, YJ-N, and AWB: conceptualization, revision, and editing of the finalized manuscript. All authors contributed to the article and approved the submitted version.

## Funding

This work was supported in part by NIH (grants R25DK099080, R25HL124208, and R25GM141507) and the Gordon and Betty Moore Foundation.

## Author Disclaimer

The opinions expressed are those of the authors and do not necessarily represent those of the NIH or any other organization.

## Conflict of Interest

AWB has received speaking fees from Purdue University and University of Arkansas for the Medical Sciences; consulting fees from LA NORC, Pennington Biomedical Research Center, and Soy Nutrition Institute Global; and grants through his institution from Alliance for Potato Research & Education, American Egg Board, Dairy Management, Inc., National Cattlemen's Beef Association, NIH/NHLBI, NIH/NIDDK, and NIH/NIGMS. He has been involved in research for which his institution or colleagues have received grants or contracts from Alliance for Potato Research & Education, Center for Open Science, Gordon and Betty Moore Foundation, Hass Avocado Board, Indiana CTSI, National Cattlemen's Beef Association, NIH/NHLBI, NIH/NIA, NIH/NIDDK, and Sloan Foundation. His wife is employed by Reckitt Benckiser. The remaining authors declare that the research was conducted in the absence of any commercial or financial relationships that could be construed as a potential conflict of interest.

## Publisher's Note

All claims expressed in this article are solely those of the authors and do not necessarily represent those of their affiliated organizations, or those of the publisher, the editors and the reviewers. Any product that may be evaluated in this article, or claim that may be made by its manufacturer, is not guaranteed or endorsed by the publisher.
